# Reply: Granisetron *vs* ondansetron: is it a question of duration of 5-HT_3_ receptor blockade?

**DOI:** 10.1038/sj.bjc.6600314

**Published:** 2002-05-03

**Authors:** R de Wit, G Stoter, A Sparreboom, J Verweij, A C de Boer, G H M vd Linden

**Affiliations:** University Hospital Rotterdam, Rotterdam Cancer Institute, Rotterdam, The Netherlands; IJsselland Hospital, Capelle aan den IJssel, The Netherlands; Albert Schweitzer Hospital, Dordrecht, The Netherlands

## Abstract

*British Journal of Cancer* (2002) **86**, 1664–1664. DOI: 10.1038/sj/bjc/6600314 www.bjcancer.com

© 2002 Cancer Research UK

## 

We appreciate Drs Blower's and Aapro's comments to our paper. The optimal design of the study obviously would have been to cross-over in both directions, but the small size of the study and its unsponsored nature did not allow for such design. We have stated in our paper that our findings merely allow to conclude that one 5HT_3_ receptor antagonist may be effective after another given at the recommended dose and schedule fails. There is no reason to believe that one 5HT_3_ receptor antagonist is better than another, we have only demonstrated that granisetron may be helpful after failure to ondansetron, like we have previously reported protection with ondansetron after failure to tropisetron (de Boer *et al*, 1995), which indicates in our opinion that there is no complete cross-resistance between these compounds.

We disagree that psychological effects have played a role in the successful cross-over in our study, as the study was double-blinded and no such effects were observed in the ‘continued with ondansetron’ group; 1 complete protection with continued use of ondansetron out of 21 patients, *vs* nine successful cross-overs, of 19 patients on granisetron (complete protection after previous acute-emesis protection failure on ondansetron).

We have written a concise report on our observations and deliberately refrained from hypothetical explanations, but we do not believe that scheduling or dosing of ondansetron may explain these results. Ondansetron was administered as is considered standard according to international consensus guidelines, which is a single dose of 8 mg prior to the chemotherapy (Roila, 1998; Gralla *et al*, 1999). Therefore we concluded that there is no complete cross resistance between 5HT_3_ receptor antagonists, and that patients who have acute emesis protection failure on one 5HT_3_ receptor antagonist should be offered cross-over to another 5HT_3_ receptor antagonist.
